# Advances in Biosensors Technology for Detection and Characterization of Extracellular Vesicles

**DOI:** 10.3390/s21227645

**Published:** 2021-11-17

**Authors:** Saif Mohammad Ishraq Bari, Faria Binte Hossain, Gergana G. Nestorova

**Affiliations:** 1 Micro and Nanoscale Systems Engineering, Louisiana Tech University, Ruston, LA 71272, USA; smi015@latech.edu; 2 Molecular Science and Nanotechnology, Louisiana Tech University, Ruston, LA 71272, USA; fbh004@latech.edu; 3 School of Biological Sciences, Louisiana Tech University, Ruston, LA 71272, USA

**Keywords:** sensors, exosomes, lab-on-a-chip

## Abstract

Exosomes are extracellular vehicles (EVs) that encapsulate genomic and proteomic material from the cell of origin that can be used as biomarkers for non-invasive disease diagnostics in point of care settings. The efficient and accurate detection, quantification, and molecular profiling of exosomes are crucial for the accurate identification of disease biomarkers. Conventional isolation methods, while well-established, provide the co-purification of proteins and other types of EVs. Exosome purification, characterization, and OMICS analysis are performed separately, which increases the complexity, duration, and cost of the process. Due to these constraints, the point-of-care and personalized analysis of exosomes are limited in clinical settings. Lab-on-a-chip biosensing has enabled the integration of isolation and characterization processes in a single platform. The presented review discusses recent advancements in biosensing technology for the separation and detection of exosomes. Fluorescent, colorimetric, electrochemical, magnetic, and surface plasmon resonance technologies have been developed for the quantification of exosomes in biological fluids. Size-exclusion filtration, immunoaffinity, electroactive, and acoustic-fluid-based technologies were successfully applied for the on-chip isolation of exosomes. The advancement of biosensing technology for the detection of exosomes provides better sensitivity and a reduced signal-to-noise ratio. The key challenge for the integration of clinical settings remains the lack of capabilities for on-chip genomic and proteomic analysis.

## 1. Introduction

**Figure 1:**
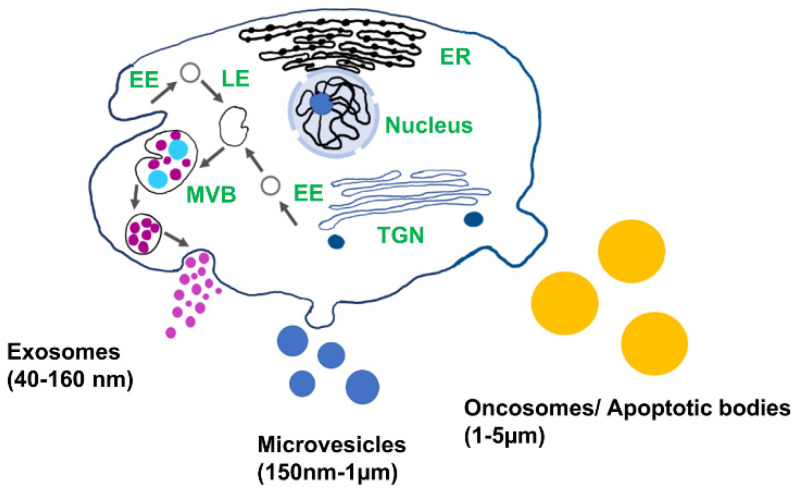
Schematic of different types of extracellular vesicles and their biogenesis: EE: early endosomes, LE: late endosomes, MVB: multivesicular body, TGN: trans-Golgi network, and ER: endoplasmic reticulum.

Extracellular vesicles (EV) play important role in cell-to-cell communication and serve as an important source of genomic and proteomic biomarkers for the early detection and diagnosis of diseases [1,2]. EVs are found in all biofluids, including blood, urine, saliva, synovial fluid, and cerebrospinal fluid, and encapsulate proteins, lipids, and nucleic acids from the cell of origin [3,4]. Depending on their biogenesis and structures, EVs are categorized as apoptotic bodies, microvesicles, and exosomes (Figure 1). Exosomes are the smallest type of EVs (40–160 nm) and are released via endocytic pathways [5]. They are considered to be a promising source of biomarkers for disease diagnosis, including cancer, diabetic cardiomyopathy, arthritis, asthma, and neurodegeneration, since their biological cargo reflects the pathophysiological condition of the host cell [6,7,8,9,10,11]. Exosomes are promising candidates as drug delivery vesicles due to their low immunogenic potential coupled with the effective protection of the nucleic acid and protein cargo from degradation. Nucleic acid and protein from the cell of origin are selectively sorted in the exosomes. The molecular cargo of the vesicle reflects the pathological condition of the parent cell and therefore provides a source of biomarkers for the early diagnosis of diseases [12]. Due to easy access to biofluids enriched in exosomes and high cell-specificity, exosomes can be used in liquid biopsy for early diagnosis.

The protein contents in exosomes include transmembrane proteins, lysosome-derived membrane proteins, lactadherin, membrane-associated proteins, GTPases, heat shock proteins, lipid-related proteins, phospholipases, tetraspanins, and proteins associated with the multivesicular body (MVs) biogenesis [13,14,15,16]. Multiple studies suggest that the proteomic analysis of exosomes can facilitate the early detection of cancer, metabolic diseases, and neurological disorders [17,18]. Therefore, the isolation of a pure exosomal subpopulation from a biological fluid is crucially important to determine their pathophysiological functions. This review provides a detailed discussion of the advantages and barriers in conventional exosome sensing techniques and the most recent advancements and challenges in biosensors for the purification and on-chip detection of exosomes. 

## 2. Exosome’s Biogenesis, Molecular Cargo, and Function

**Figure 2:**
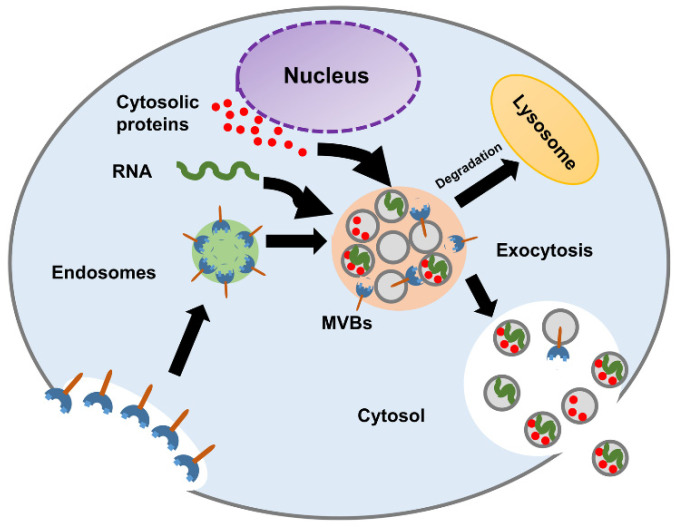
Schematic diagram of exosome biogenesis and molecular composition.

The biogenesis of exosomes starts in early endosomes. Matured or late-endosomes move to the cytoplasm, change their tube-like shape into a spherical one, and form the multivesicular bodies (MVBs). Inward budding results in the progressive accumulation of intraluminal vesicles (ILVs) inside the MVB. The development of the multivesicular endosomes can follow either of the following pathways, as shown in Figure 2:

- MVBs fuse with lysosomes and degrade their content.
- MVBs fuse with the plasma membrane and release their content into the extracellular space.

For the first pathway, the MVBs are hydrolyzed by the lysosomes. When the MVBs fuse with the plasma membrane, the exosomes are released into the extracellular space [19]. Studies have indicated that the formation of ILVs and the process of exosome release are mediated by tetraspanins (CD9, CD63, CD81), glycan modification, and Rab guanosine triphosphatases (RAB 27A, RAB 27B, RAB 31, and RAB 11) [1,11,20,21]. The complex underlying biology of exosome formation and what factors govern the transition of the MVBs to the exocytic pathway or the degradative pathway remain to be further characterized [4].

Exosomes contain a diverse cargo that includes proteins, DNA, mRNA, non-coding RNA, tRNA, DNA, and lipids [21]. The proteomic content comprises exosomal marker proteins (TSG101, HSP90β, HSC70, Alix), tetraspanins (CD9, CD63, CD81, CD82), and cell-specific proteins (ex. EpCAM) [21,22]. The exosomal marker proteins are found in the exosomes regardless of the cell of origin [23,24]. The transmembrane proteins belong to the tetraspanin family and are enriched in the exosomes [25]. However, the tetraspanin proteins are also available in MVs and apoptotic bodies [26], as are the EVscontain miRNA, mRNA, tRNA, nucleic acids, DNA, and lipids [27,28,29,30,31,32,33]. The lipid contents in exosomes include saturated fatty acids, cholesterol, sphingomyelin, and phosphatidylserine [34]. 

EVs mediate cell-to-cell communication via the transfer of genomic or proteomic content from the donor to the recipient cell [35]. The cell can alter the exosomal cargo in response to the changes in the extracellular environment. Malignant cells secrete EVs that facilitate metastasis, and EVs influence the immune response [36]. Due to their involvement in pathological processes exosomes, can provide diagnostic biomarkers and deliver therapeutic agents. The further development of exosome-based diagnostic and drug delivery platforms requires rapid and specific isolation techniques. For example, the cerebrospinal fluid contains EVs that carry vital information about the function of the central nervous system, and methods for purification that can noninvasively access and preconcentrate the vesicles could provide technology for the early diagnosis of neurological disorders [37]. Therefore, the development of a highly selective method for EV separation and characterization is important for the accurate identification of biomarkers and the selective drugs or nucleic acid delivery to the target cells. 

## 3. Conventional Methods for Isolation and Detection of Exosomes

The main methods for exosome isolation are based on the properties of the vesicles such as size, protein surface markers, and density. These include ultracentrifugation [38], immunoaffinity-based techniques [39,40,41,42], size-exclusion chromatography [43,44], and polymer-based precipitation [45]. The advantages and disadvantages of each technique are presented in Table 1. 

The conventional methods for the detection and characterization of exosomes include flow cytometry, ELISA, tunable resistive pulse sensing (TRPS), dynamic light scattering (DLS), and nanoparticle tracking analysis (NTA). Flow cytometry is based on intersecting a laser beam with fluorescence-labeled exosomal particles and provides a high throughput multiplex analysis of surface markers [53]. Despite these advantages, the technique provides low sensitivity and resolution [54]. ELISA is based on antibody-mediated detection and the quantification of exosomal particles and is one of the most commonly used techniques for the detection of EVs [55]. Digital PCR provides excellent sensitivity for a low volume of samples [56,57]. TRPS is based on the principle that the movement of the non-conductive components in an electrolyte solution can cause a change in the electrical impedance relative to the particle concentration, size, and surface charge [58]. TRPS provides accuracy in determining the size, concentration, and surface charge of exosomes [59,60]. Both DLS and NTA assess the Brownian motion of the exosomes to measure the size and concentration of the nanoparticles [61]. While the DLS method analyzes the relative change in the intensity of the scattered light induced by the Brownian motion of the suspended nanoparticles, e.g., exosomes, NTA implements the Stokes–Einstein equation to calculate the hydrodynamic diameter of the nano components [62,63]. Due to their capabilities of discerning nanoparticles of various sizes ranging from 1 to 1000 nm, both DLS and NTA have been widely used for the detection and quantification of exosomes. However, the accuracy of the results can be reduced for samples that are prone to aggregation [61]. While all of these techniques are successfully used in research settings, the requirements of sophisticated instruments, the implementation of separate techniques for isolation and analysis, and the interference of other types of EVs create challenges for application in clinical settings [64]. 

## 4. Sensors for Detection and Molecular Characterization of Exosomes 

Tremendous progress has been made in the area of biosensor technology for the detection, quantification, and analysis of exosomes. Lab-on-a-chip biosensing offers multiple advantages over the conventional techniques that include low sample volume input, multiplexing, cost-effectiveness, and precise fluidic control [65,66]. Microfluidics provides single sample input–output, reduced cross-contamination, more efficient isolation, and increased sensitivity of quantification for applications in clinical settings [67]. To simplify and automate the purification and characterization of exosomes and other EVs, the conventional methods discussed in the previous section have been integrated with the lab-on-a-chip platforms. The biophysical characterization and separation of the heterogeneous exosome population have been successfully performed in microfluidic devices using the asymmetric flow field-flow fraction (AF4). The EVs were separated based on their density and hydrodynamic properties [68]. Nanoporous membranes were integrated into a microdevice (ExoTIC) that enabled the efficient, size-dependent separation of EV for subsequent molecular analysis. This platform provides a higher yield than conventional ultracentrifugation and polymer precipitation techniques [69]. An integrated centrifugal microfluidic platform enabled the efficient enrichment (greater than 95%) of EV and the subsequent on-chip ELISA detection of surface markers [70]. A ZnO nanowire-functionalized PDMS microfluidic platform was successfully used for the enrichment of urine-derived EVs [71]. Size-selective EV separation was demonstrated in a microchip (ExoSMP) by combining membrane filtration and electrophoretic force. Separation of different subgroups can be performed by altering the pore size of the membrane [72]. The following sections discuss the most recent advances in biosensor technology for EV detection and molecular characterization.

### 4.1. Fluorescence-Based Sensors

**Figure 3:**
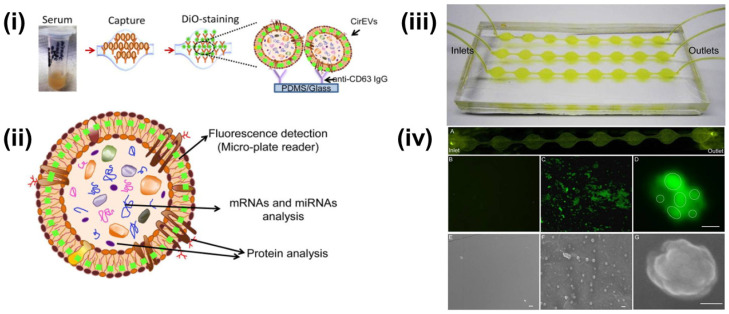
(**i**) Schematics of CD63 enrichment and membrane-specific DiO staining of exosomes using ExoChip platform. (**ii**) The fluorescent intensity of the DiO stained exosomes is measured using a fluorescent plate reader, while the genomic and the proteomic content can be characterized using Western blot or RT-qPCR analysis. (**iii**) Model of PDMS-based ExoChip. The device consists of multiple, connected circular chambers with a diameter of 5 mm and 100 µm in height. (**iv**) Microscopy visualization of the exosomes and the ExoChip channel. (**A**) Fluorescence image of DiO stained exosomes immobilized to the lower surface of the device. Fluorescent image of the control chamber (**B**) and anti-CD63 coated chamber with captured exosomes (**C**), 400×. (**D**) A confocal microscopic image of a cluster of exosomes (bar = 2 μm). Electron micrograph (EM) images of control ExoChip (**E**) and anti-CD63 functionalized ExoChip (**F**) (bar = 500 nm). (**G**) EM image of a cluster of exosomes (bar = 100 nm). Reprinted (adapted) with permission from Kanwar et al., 2014. Copyright 2014, Royal Society of Chemistry.

Multiple researchers have reported upon the fluorescent-labeling of exosomes combined with different isolation approaches. Kanwar et al. reported the on-chip exosome isolation and analysis platform, ExoChip, which employed the CD63 antibody-mediated isolation of exosomes from blood serum (Figure 3). Subsequently, the captured exosomes were stained with DiO fluorescent dye followed by quantification using a plate reader [73]. 

A homogeneous magneto-fluorescent exosome (hMFEX) nanosensor has been developed for rapid tumor-derived exosomes analysis [74]. This platform was able to detect tumor-derived exosomes with high specificity and sensitivity. The limit of detection was 6.56 × 10^4^ particles μL^−1^, demonstrating the potential clinical diagnostic efficacy [74]. Quantification and differentiation between a normal and metastatic sentinel lymph node (SLN)-derived exosomes captured using fluorescent silicon nanoparticles-based exosome probes (SiNPs@EXO) have successfully been demonstrated [75]. To study the exosomes derived from mouse breast cancer (4T1) and human embryonic kidney (HEK293T) cells, 1,8-napthalimide fluorophores were used. Exosomes derived from 4T1 cells showed higher fluoresce intensity than the negative control (HEK293T). Fluorescence analysis depicted that the fluorescence signals in metastatic SLNs reached a peak within 30 min and stayed for up to 3 h, whereas normal SLNs attained the peak in about an hour followed by a sudden decrease in the signal [75]. This integrated approach can be used for predicting lymphatic metastasis via the detection of SLN-derived exosomes. 

### 4.2. Colorimetric Sensors

**Figure 4:**
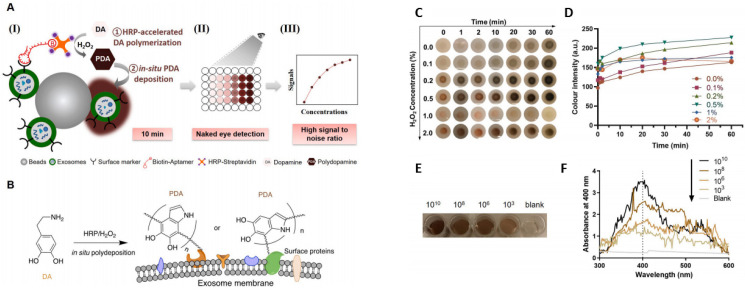
(**A**) (**I**) Exosomes were anchored on sulfate/latex beads by aldimine condensation followed by binding to a CD63-specific aptamer conjugated to biotin. Incubation with streptavidin-conjugated HRP converted colorless dopamine (DA) into brown-black colored polydopamine (PDA). (**II**) The color change is proportional to exosome concentrations. (**III**) The absorbance signals of the reaction can also be measured at 400 nm. (**B**) Schematics of the deposition of PDA onto the exosome membrane. (**C**) The color change of PDA deposition at the target site of HRP at different H_2_O_2_ concentrations. (**D**) The change in the color intensity is a function of time and H_2_O_2_ concentration. (**E**) Images of exosome samples after color development in the ExoAptaSensor. (**F**) Absorbance peak at 400 nm correlates with exosome concentrations. Reprinted from Development of a simple, sensitive, and selective colorimetric aptasensor for the detection of cancer-derived exosomes, Volume 169, Xu et al., 2020a, Page No. 112576, Copyright 2020, with permission from Elsevier.

On-chip colorimetric detection has been employed by many groups to quantify and characterize exosomes. Vaidyanathan et al. reported a multiplexed microfluidic platform with antibody-functionalized electrodes specific for capturing exosomes. The approach is based on the alternating current electrohydrodynamic (ac-EHD)-induced surface shear force (also known as nano shearing) that stimulates the fluid flow within a few nanometers of the electrodes. Absorbance measurement of the colorimetric solution was used to detect and quantify exosomes with high specificity. This platform showed superior sensitivity (2760 exosomes µL^−1^) compared to other fluid dynamic-based approaches [76]. A sensitive and selective colorimetric aptasensor was successfully implemented for the detection of cancer-derived exosomes. The chromogenic signal was produced by the polymerization of horseradish peroxidase (HRP)-accelerated dopamine (DA) and the in situ deposition of polydopamine (PDA) [77]. The target exosomes were first isolated by latex beads followed by bio-recognition using a specific CD63 aptamer, which was conjugated to horseradish peroxidase (HRP) through biotin-streptavidin binding [77] (Figure 4). Colorimetric detection was completed in 10 min via enzymatic catalysis, which produced dark-colored polydopamine (PDA) from colorless dopamine (DA). The color depth correlated to the CD63 amount, and the reported limit of detection (LOD) was 7.7 × 10^3^ particle mL^−1^, increasing the LOD by 3–5 orders of magnitude from conventional Dot-blot methods [77]. 

A highly sensitive plasmonic colorimetric biosensor for exosome quantification based on a two-step sensing technique was reported. The technique employed an exosome-induced competitive reaction and etching of gold nanobipyramid@MnO_2_ nanosheet nanostructures (Au NBP@MnO_2_ NSs) [78]. A competitive reaction induced by exosomes translated the signal of exosomes into the amount of alkaline phosphatase, which simplified the experimental process and amplified the signal. The refractive index of Au NBPs was increased via the etching of the Au NBP@MnO_2_ NSs by ascorbic acid. The combination of excellent refraction and signal amplification provided a limit of detection of 1.35 × 10^2^ particles μL^−1^, providing superior exosome detection sensitivity than previously reported colorimetric methods [78].

### 4.3. Magnetic Sensors

**Figure 5:**
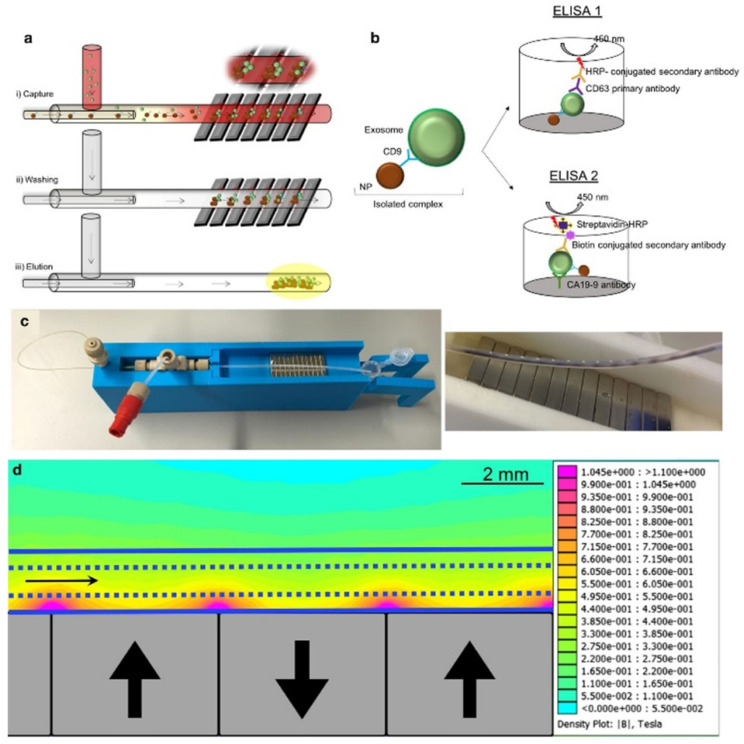
(**a**) Exosomes are captured by antibody functionalized nanoparticles (Fe_3_O_4_-EDC-NHS-NP: anti CD9). (**b**) ELISA was performed to quantify exosomes and to measure CA19-9 levels. (**c**) Experimental setup and capture of the magnetic particles in the channel. (**d**) Simulation of the magnetic gradient. Reproduced with permission from Sancho-Albero et al., 2020 under Creative Commons Attribution License (CC BY 4.0), Copyright 2020, Springer Nature.

Because of their small size, exosomes interfere with the detection capabilities of traditional nuclear magnetic resonance (NMR). Micro-nuclear magnetic resonance (µNMR) was developed for capturing small particles such as exosomes [79]. Shao et al. developed a microfluidic-based µNMR device for the detection and differentiation of multiforme glioblastoma EVs from non-tumor host cell-derived EVs. The vesicles were isolated using immunomagnetic nanoparticles (IMNPs) followed by the filtration of the super magnetic IMNP–exosome complexes. This approach resulted in the excellent reproducibility and accuracy of EV quantification and protein characterization [80]. Sancho-Albero et al. reported a continuous-flow microfluidic device for the isolation and analysis of whole blood exosomes derived from pancreatic cancer (PC) patients via CD9-mediated magnetic capture (Figure 5). The specificity of the technique was validated via the ELISA analysis of exosomal Ca19-9 levels in PC patients and healthy individuals. This platform can be used for the early detection and assessment of PC progression using exosome analysis [81]. 

### 4.4. Surface Plasmon Resonance (SPR) Sensors

**Figure 6:**
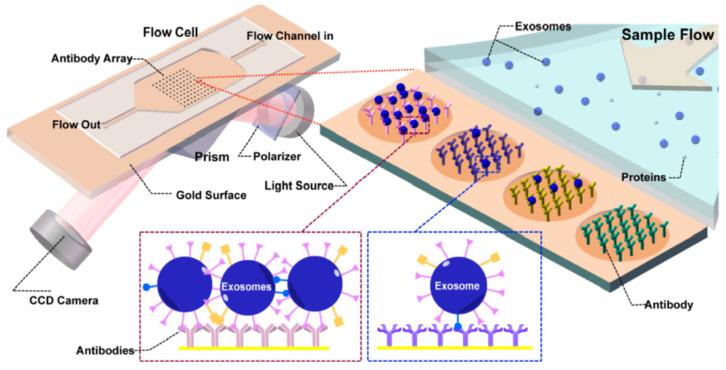
Principle of operation of SPR sensor incorporating an antibody array specific to exosomes transmembrane proteins. The binding of the exosomes causes a change in the refractive index of the laser that is detected by the CCD camera. Reproduced with permission from Zhu et al., 2014 (article link: https://pubs.acs.org/doi/10.1021/ac5023056 (accessed on 30 September 2021), Copyright 2014, American Chemical Society. Any future use of this content is subjected to permission from ACS Publications.

Surface plasmon resonance (SPR) is defined as the resonant oscillation of the electrons stimulated by the incident light at the interface between a negative and a positive dielectric constant material. This oscillation enables the sensitive detection of change in the boundary conditions, which is employed for the detection of adsorption of biomolecules to the surface [82,83]. Microfluidic-based SPR is an emerging method of exosome isolation due to its cost-effectiveness, portability, and the ability of fast and label-free detection. An on-chip SPR sensor was developed for the isolation and quantification of tumor-derived exosomes. The gold surface was functionalized with antibodies specific to exosome surface markers, and the refractive index changed upon the binding of the vesicles to the capture antibodies. The antibody array enabled the multiplex analysis of the surface markers in a single sample (Figure 6) [84]. 

W. Chen et al. reported a label-free real-time surface plasmon resonance imaging (SPRi) biosensor based on the hydrogel-gold nanoparticles supramolecular sphere (H-AuNPs). This approach was successfully used for the detection and quantification of prostate cancer cell-derived exosomes. The localized surface plasmon resonance of AuNPs and the signal amplification effect of the mass cumulative multi-layered porous hydrogels enhanced the sensitivity of the platform. The sensor has a limit of detection of  10^5^ particles mL^−1^ [85]. The SPRi-based quantification correlated with the tumor-derived prostate-specific antigen (t-PSA) values measured via clinically validated chemiluminescence immunosensors. The microfluidic biosensor was successfully employed for the analysis of human serum samples and could have future applications in clinical settings.

### 4.5. Electrochemical Sensors

**Figure 7:**
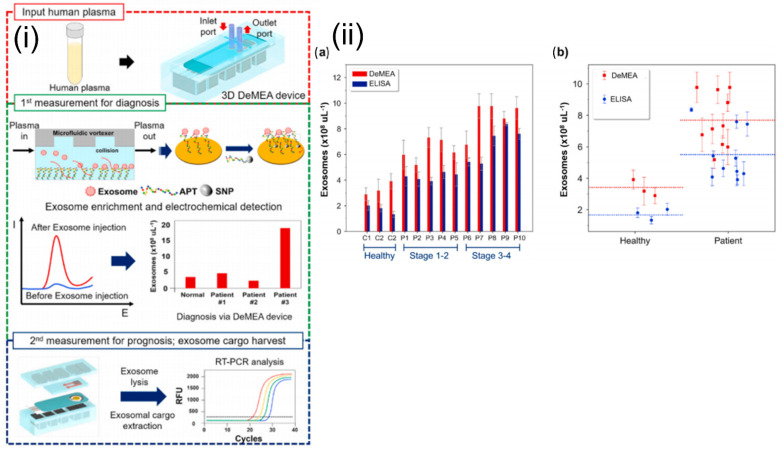
(**i**) Schematics and experimental workflow of the microfluidic platform with electrochemical aptasensor (DeMFA) for the quantification and cargo analysis of exosomes. (**ii**) DeMEA and ELISA quantification of exosomes derived from plasma samples of healthy and MCF-7 cancer cells represented in: (**a**) bar graph, (**b**) scatter graph (n = 3).Reprinted from Detachable microfluidic device implemented with electrochemical aptasensor (DeMEA) for sequential analysis of cancerous exosomes, Volume 169, Kashefi-Kheyrabadi et al., 2020, Page No. 112622, Copyright 2020, with permission from Elsevier.

Electrochemical sensors measure the electric current generated from oxidative or reductive reactions. This approach can be easily integrated with a microfluidic platform and enables highly sensitive biomolecular detection [86]. Zhou et al. reported an aptamer-based electrochemical biosensor for the quantitative detection and analysis of exosomes in a microfluidic device. The aptamers specific to the exosome-specific tetraspanin protein, CD63, were immobilized on gold electrodes. The concentration of the exosomes was inversely proportional to the signal generated due to the binding of methylene blue-labeled probe strand and the CD63 aptamer. Signal output decreased in the presence of the exosomes, as they displaced the probe strands. This reported biosensor was able to detect 10^6^ per mL^−1^ exosomes, representing a 100-fold improvement in sensitivity over commercial ELISA assays [87]. A detachable microfluidic device with an integrated electrochemical aptasensor was successfully used for the detection and genomic characterization of breast cancer-derived exosomes (Figure 7). An aptamer specific to epithelial cell adhesion molecules was immobilized onto the gold-plated electrode, and a microfluidic vortexer was integrated using 3D printed magnetic housing. The hydrodynamically generated transverse flow increased the rate of collisions between the exosomes and sensing surface. The reported platform demonstrated ultra-high sensitivity (17 exosomes µL^−1^) over a wide dynamic range (1 × 10^2^ to 1 × 10^9^) exosomes μL^−1^ [88].

### 4.6. Immunoaffinity Sensors

**Figure 8:**
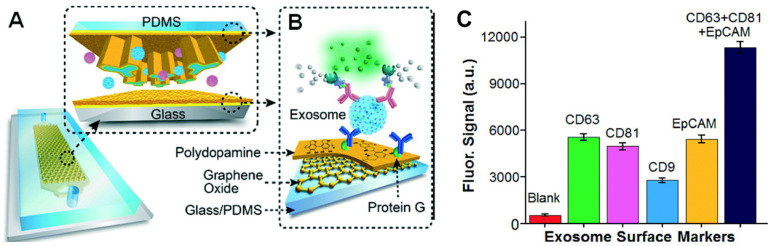
(**A**) Schematics of microfluidics platform based on graphene oxide/polydopamine nano-interface (nano-IMEX) for exosome immunocapture; (**B**) detection of exosomes surface markers via sandwiched ELISA. (**C**) Exosome surface marker profiling of COLO-1 cell exosomes (10^6^ μL^−1^) captured by CD81 mAb. Reproduced with permission from Zhang et al., 2016 under Creative Commons Attribution Non-Commercial 3.0 Unported License, Copyright 2016, Royal Society of Chemistry.

Immunoaffinity-based approaches are employed for the isolation and quantification of exosomes that express specific surface markers. The specificity of the antibody and the degree of nonspecific binding of the exosomes to the surface affect the purity of the exosomal subpopulation. The antibody-based isolation of exosomes using the anti-CD63 antibody was successfully performed on a microfluidic device with herringbone groves that ensured efficient mixing, manipulation, and separation of the fluid and the vesicles [89]. Kanwar et al. developed an anti-CD63 antibody functionalized PDMS microfluidic chip for the isolation of exosomes from serum samples [73]. A glass-PDMS device coated with graphene oxide and polydopamine was employed for the selective capture of CD81 expressing exosomes from plasma samples (Figure 8). This nanostructured interface improved the efficiency and reduced nonspecific binding to the surface of the device while providing a 4 log dynamic range of detection. The platform was successfully employed for the detection and discrimination of exosomes derived from ovarian cancer patients and healthy individuals using 2 µL of serum [90]. Zhang et al. developed a herringbone mixer-based microfluidic chip that was able to directly separate exosomes from plasma using an immunoaffinity-based approach. This method enabled the successful enrichment of pancreatic cancer expressing Glypican-1 exosome subpopulations with 75% efficiency [91]. Recently, another research group demonstrated the feasibility of a solid-phase, microprobe-based technology for CD63-specific exosome purification that can further be integrated with a microfluidic platform for high-throughput and integrated omics analysis [42]. The ExoPRIME tool provides a Precise, Rapid, Inexpensive, Mild (non-invasive), and Efficient (i.e., PRIME) alternative for exosome isolation and analysis from both conditioned astrocyte media (CAM) and enriched exosome suspension (EXO). The results indicated that the reduced temperature with extended incubation times ensured high probe loading capacity (24 × 10^6^ exosomes per microprobe). The probe (3 mm × 130 µm) captured a sufficient number of vesicles for subsequent genomic and proteomic analysis. The RNA capture efficiency was 0.54 ng probe^−1^ and 0.30 ng probe^−1^, respectively, for the EXO and CAM samples. The reported protein loading capacity was 940 ng probe^−1^ and 728 ng probe^−1^, respectively, for the EXO and CAM samples [42]. The same research group has also designed and mathematically identified the optimal design parameters of a microfluidic cell co-culture device with a pneumatically controlled valve that can be integrated with the ExoPRIME capture method for genomic and transcriptomic analysis of exosomes derived from the central nervous system and astrocytes [92]. 

A summary of the lab-on-a-chip methods used for the isolation of exosomes is presented in the following Table 2:

## 5. Conclusions

This article summarizes the biological functions of exosomes and their functional roles as disease biomarkers and prospective applications for point-of-care diagnostics. The conventional isolation and detection methods and the recent advances in sensor technology have been discussed, including their advantages and disadvantages. Although on-chip biosensing provides a rapid and more accurate quantification and analysis of exosomes from various biofluids compared to conventional techniques, the challenge of integration with high-throughput genomic and proteomic instrumentation remains. Due to the heterogeneous nature of EVs, it is often very difficult to obtain highly pure exosomes that result in lowering the accuracy of the detected biomarkers. The preparation of a pure EV population is an important starting point in the development of point-of-care tests for disease diagnosis and for the development of novel therapeutic agents. Because of the ongoing research and development efforts to integrate all of the sample processing and analysis steps in a single platform, it can be anticipated that in the future, exosome isolation and detection could be performed in a single integrated microfluidic device for the application in point-of-care diagnostics. 

## Figures and Tables

**Table 1:** Summary of a comparative study of existing conventional exosome isolation techniques.

Isolation Technique	Isolation Principle	Appraisal Parameters			Advantages	Disadvantages	References
		Time	Yield	Purity			
Ultracentrifugation	Density-based	*	*	**	The gold standard	Time-consuming; low yield; moderate purity;	[38,46]
Immunoaffinity-capture	Affinity-based	**	**	***	Highly specific and pure	Expensive; strict pH condition; constrained use	[40,41]
Size-exclusion chromatography	Vesicles Size	**	**	**	Simple, rapid, moderate yield	Poor specificity; scaling problems	[44,47,48]
Polymer-based precipitation	Surface charge-based	*	***	*	Simple and user-friendly, high yield	Expensive; low specificity; poor purity; scaling problems	[22,49]
Ultrafiltration	Molecular weight and size-based	**	*	**	Simple, no specific instrument	Clogging; low yield, low specificity, time-consuming	[50,51,52]

Appraisal parameters are categorized by ***, **, and * imply very good, moderate, and poor performances, respectively.

**Table 2:** Summary of the on-chip EVs detection techniques and reported limits of detection.

Sensor Type	Isolation Principle	Minimum Detection Limit	References
Fluorescence	Quantification of fluorescence signal	6.56 × 10^7^ EVs mL^−1^	[74]
Colorimetric	Absorbance measurement of the colorimetric solution	1.35 × 10^5^ EVs mL^−1^	[78]
Magnetic	Nuclear magnetic resonance	4.39 × 10^3^ EVs mL^−1^	[93]
Surface Plasmon Resonance (SPR)	Resonant oscillation of the electrons stimulated by the incident light at the interface between a negative and a positive dielectric constant material	10^5^ EVs mL^−1^	[85]
Electrochemical	Measurement of the electric current caused by the oxidation or reduction reactions	10^6^ EVs mL^−1^	[87]
Immunoaffinity	Affinity-based isolation	24 × 10^6^ EVs mL^−1^	[42]

## Data Availability

Not applicable.
